# The Antimicrobial Properties of Cannabis and Cannabis-Derived Compounds and Relevance to CB2-Targeted Neurodegenerative Therapeutics

**DOI:** 10.3390/biomedicines10081959

**Published:** 2022-08-12

**Authors:** HeeJue Hong, Lucy Sloan, Deepak Saxena, David A. Scott

**Affiliations:** 1 Oral Immunology and Infectious Diseases, University of Louisville School of Dentistry, Louisville, KY 40202, USA; 2 Pharmacology and Toxicology, University of Louisville School of Medicine, Louisville, KY 40292, USA; 3 Molecular Pathobiology, New York University College of Dentistry, New York, NY 10010, USA

**Keywords:** antibiotic resistance, cannabis, cannabinoids, CB2 receptors, microbial infection, novel antimicrobials

## Abstract

Cannabinoid receptor 2 (CB2) is of interest as a much-needed target for the treatment or prevention of several neurogenerative diseases. However, CB2 agonists, particularly phytocannabinoids, have been ascribed antimicrobial properties and are associated with the induction of microbiome compositional fluxes. When developing novel CB2 therapeutics, CB2 engagement and antimicrobial functions should both be considered. This review summarizes those cannabinoids and cannabis-informed molecules and preparations (CIMPs) that show promise as microbicidal agents, with a particular focus on the most recent developments. CIMP–microbe interactions and anti-microbial mechanisms are discussed, while the major knowledge gaps and barriers to translation are presented. Further research into CIMPs may proffer novel direct or adjunctive strategies to augment the currently available antimicrobial armory. The clinical promise of CIMPs as antimicrobials, however, remains unrealized. Nevertheless, the microbicidal effects ascribed to several CB2 receptor-agonists should be considered when designing therapeutic approaches for neurocognitive and other disorders, particularly in cases where such regimens are to be long-term. To this end, the potential development of CB2 agonists lacking antimicrobial properties is also discussed.

## 1. Introduction

In the brain, CB2 is expressed by microglia, astrocytes and neurons, while CB2-agonism may induce cell-specific events [1]. CB2-engaging molecules, including cannabinoids, have received considerable attention as potential therapeutic and/or preventive agents for neuropathic pain, neuroinflammation, variant dementias, and other neuropathies [1,2,3,4]. As reviewed elsewhere, CB2 manipulation has shown preclinical promise in treating Alzheimer’s disease (e.g., reduced Tao phosphorylation; protection against Aβ-induced injury and suppressed microglia activation), Parkinson’s disease (e.g., prevention of neurodegradation; reduced neuroinflammation), Huntington’s disease (protection of striatal neurons; suppression of CNS inflammation) and other neurodegenerative disorders [3]. Cannabis and cannabinoids have been reported to provide motor symptom relief and to provide respite from behavioral and psychological dementia symptoms [2]. However, multiple phytocannabinoids and other cannabis-related molecules, many of which are CB2 agonists, have been reported to act as potent antimicrobial agents which may lead to unintended health consequences, particularly during chronic medicinal treatments. As these antimicrobial agents are widely varied, we use the phrase cannabis-informed molecules and preparations, or CIMPs, to include cannabis, phytocannabinoids, cannabis-informed molecules and cannabis-informed preparations.

With the global growth in the number of jurisdictions where recreational and/or medicinal cannabis is decriminalized, an accompanying increase in cannabis consumption may be considered likely. While any relationships between cannabis use and altered risk to infectious diseases remain to be more definitively elucidated, there has been a recent resurgence of interest in the potential of cannabis and cannabis-derived compounds as novel antimicrobial agents. This anti-microbial potential needs to be considered carefully when developing medicinal CB2-engaging CIMPs for other purposes, such as protection against neurodegeneration. The recent upturn in attention in CIMPS as antimicrobials is largely due to the major contemporary crisis of the reducing efficacy of the available antimicrobial armory against important bacterial pathogens, including, but by no means limited to, *Escherichia coli*, *Staphylococcus aureus*, *Klebsiella pneumoniae*, *Streptococcus pneumoniae*, *Acinetobacter baumannii* and *Pseudomonas aeruginosa*. The importance of this public health emergency is best accentuated by the recent estimate of 4.95 million global deaths associated with bacterial antimicrobial resistance in 2019 alone [5].

Ongoing research on CIMPs as antimicrobial agents has been largely predicated by, primarily, Eastern European literature from the mid-twentieth century [6,7,8,9,10]. There have been a number of recent reviews published that highlight the potential of CIMPs as novel anti-microbial agents [8,11,12,13,14,15,16,17]. With this recent revitalization of the field, the purpose of the present article, then, is (a) to update and augment the rapidly expanding literature, with a particular focus on the most recent developments; (b) to compare variant CIMP antimicrobial activities, where quantified; (c) to address specific mechanisms of CIMP–microbe interactions in greater depth; (d) to highlight the many major knowledge gaps alongside barriers to translation; (e) to discuss the potent antimicrobial activities of some CB2 receptor agonists which should be considered when designing therapeutic treatments for neurocognitive disorders; and, particularly, (f) that non-microbicidal CB2 agonists could be preferentially designed, engineered and utilized for long-term anti-neuropathic purposes, where appropriate.

## 2. The Antimicrobial Properties of Cannabis and Cannabis-Derived Compounds

### 2.1. Infectious Diseases in Cannabis Users

While tobacco smoking has been established as a risk factor for a plethora of infectious diseases [18], including periodontitis, otitis media, tuberculosis, pneumonia, gonorrhea and meningitis, related epidemiological studies on the potential association between cannabis consumption and human microbial illnesses are surprisingly scarce.

The limited data available, however, suggest that cannabis consumption may be associated with alterations to mucosal microbial profiles [19]; the development of a diverse non-*Lactobacillus*-predominant vaginal microbiota [20]; an altered oral microbiome with an enhanced *Streptococcus* and *Actinomyces* and depressed *Neisseria* content [21]; risk of sexually transmitted disease in men [22]; seropositivity for hepatitis C virus [23]; and increased risk of fungal infections [24]. Moreover, Johnson et al. have recently reported that environmental exposure to cannabis smoke in children increases the likelihood of viral respiratory diseases [25]. Therefore, there is a clear need for a greater understanding of any potential relationships between cannabis use and microbial-induced diseases.

What is becoming more apparent, however, is that CIMPs seem to exert often potent antibiotic-like activities against a large number of highly varied microbes. The major *Cannabis sativa*-derived cannabinoids, particularly cannabidiol (CBD), Δ9-tetrahydrocannabinol (THC), and cannabinol (CBN), have received the most attention, although over 120 phytocannabinoids are known, falling into 11 major structural groupings [14]. Contemporary publications addressing CIMPs as antimicrobials are summarized in Table 1, Table 2, Table 3, Table 4 and Table 5. Please note that studies quantifying the anti-microbial activities of cannabis-informed molecules from 218-2022 are summarized. Negative data are not presented. Please see the original publications for this information. As is apparent from Table 1, Table 2 and Table 3, most recent research has focused on the antibacterial properties of cannabis-related molecules, with relatively little attention paid to potential anti-mycotic (Table 4) and anti-viral (Table 5) agents. That said, the potential anti-SARS-CoV-2 activities of CIMPs are intriguing if, perhaps, contentious.

### 2.2. Antibacterial Properties of Cannabis and Cannabis-Derived Compounds

The most recent studies on the antimicrobial properties of CIMPs, where minimal inhibitory concentrations or related measures have been determined, are summarized in Table 1, Table 2, Table 3, Table 4 and Table 5. There is a growing body of evidence that such formulations have impressive bactericidal activities against some important, therapeutically problematic Gram-negative pathogens, such as *E. coli* and *P. aeruginosa* (Table 1), methicillin-resistant *S. aureus* strains (MRSA; Table 2) and other Gram-positive bacteria (Table 3). A summation of the anti-*S. aureus*-specific properties of CIMPs has recently been published [52].

In addition to the data presented in Table 1, Table 2 and Table 3, acid-fast bacteria have also been considered while there are multiple other recent studies that report anti-microbial activities associated with CIMPS, but where MICs were not presented. The minimum inhibitory concentrations of CBD for *Mycobacterium tuberculosis* H37Rv ATCC 27294 and *M. tuberculosis* CF86 have been reported as 12.5 and 25 mg/mL, respectively [31]. Zheljazkov et al. have reported that essential oils from *Cannabis sativa* L. cv. Novosadska exhibit antimicrobial activity against *S. aureus* CCM 4223, *Salmonella enterica* CCM 3807, *Yersinia enterocolitica* CCM 5671 and *P. aeruginosa* CCM 1959 [53]. Pasquali et al. have reported that multiple-resistant and antibiotic-sensitive strains of *Salmonella* Typhimurium (ST208, ST63) and *E. coli* (ATCC 25922, EC135), but not *Listeria monocytogenes*, are susceptible to killing by CBD and essential oils from *C. sativa* L. Futura 75 [54]. Gildea et al. have reported that CBD is active against *Salmonella newington* as well as *S*. Typhimurium [44]. The phytocannabinoid, cannabichromenic acid (CBCA), has been reported to exert potent antibacterial activity against clinical strains of *Enterobacter faecalis* and both methicillin-resistant and sensitive strains of *S. aureus*. This occurs in a manner that is independent of bacterial cell density and occurs more rapidly than the action of the critical antibiotic, vancomycin [55].

Briefly, it should be noted that there are non-*C. sativa* plants among the *Cannabaceae* family that are, or have been, used medicinally around the world but have received only minor attention from the scientific community [56]. For example, extracts of flower heads of *Trema orientalis*, which is widely distributed in tropical regions of Asia, have been reported to exhibit antibacterial activities against methicillin-resistant *S. aureus* (ATCC 43300), *P. aeruginosa* (ATCC 27853), and *Acinetobacter baumannii* (ATCC 19606) with MICs of 64–125 µg/mL, 31–64 µg/mL and 31–64 µg/mL, respectively [56]. Such extracts were shown to contain THC, CBN and, to a lesser extent, CBD. While there is a growing number of studies on cannabis-informed antimicrobials, the field can still be considered to be in its infancy, albeit one with renewed recent interest.

CBD has been shown to exhibit anti-biofilm efficacy against *S. aureus* ATCC 25923, a methicillin-sensitive (MSSA) strain, and ATCC 43300, an MRSA strain, with minimum biofilm eradication concentrations of 1–2 μg/mL and 2–4 μg/mL, respectively [11]. Farha et al. have shown that multiple cannabinoids employed at 2 µg/mL are potent inhibitors of *S. aureus* biofilm formation, with cannabigerol (CBG) at 4 µg/mL seemingly able to disperse preformed biofilms and rapidly kill persister cells [12]. CBD and CBG have also been shown to prevent biofilm formation by the related, cariogenic bacterium, *Staphylococcus mutans* UA159 [57,58].

In a model of contact lens infection, Di Onofrio et al. reported that CBD may have functionality as an adjunctive agent for the inhibition of *P. aeruginosa* biofilm formation [33], while essential oils from hemp have been reported to reduce *L. monocytogenes* biofilm-formation capability [42]. Aqawi et al. [59] reported that cannabigerol (CBG) slowed planktonic growth and inhibited *Vibrio harveyi* quorum sensing, in a manner not rescued using exogenous autoinducers, and that CBG also reduced biofilm formation and bacterial motility.

Moreover, Silvestri et al. have shown that CBD administration, with or without adjunctive fish oil, alters the fecal microbiota in a dextran sulphate sodium-induced murine colitis model [60]. Similarly, Skinner et al. have determined that a CBD-rich hemp extract, delivered in the diet of normal black mice, induced microbial fluxes in the gut, including alterations in the abundance of *Akkermansia* *muciniphila*, an intestinal probiotic species [61]. Lastly, Newman et al. have previously shown that cannabis can exert a profound influence on mucosal microbial communities by establishing that cannabis-related microbial fluxes occur at common sites of head and neck squamous cell carcinoma, specifically the tongue and oral pharynx [19].

Clearly, there is a need for further research into the therapeutic potential of cannabis and CIMPs against complex microbial communities. In the meantime, the reported antimicrobial activities of phytocannabinoids should be considered when formulating neurological therapies with related CB2-engaging agents, especially when intended for long-term use.

### 2.3. Anti-Fungal Activities of Cannabis, Cannabis-Derived Compounds

Earlier work suggested that cannabis extracts have anti-mycotic potential, e.g., [62,63]. Contemporary studies of the anti-fungal properties of cannabis-related molecules, however, are limited in number. Those in which MIC, or equivalent quantitative indices, have been reported are summarized in Table 4. Due to the likely increase the use of cannabis and cannabis-informed therapeutics, which include CB2 agonists, it is important to further our understanding of how cannabis interacts with fungi.

The potential for treatment of *Candida albicans* infections is of particular interest, with CBD exhibiting anti-planktonic and anti-biofilm activities against this particular yeast [27,46]. Feldman et al. have reported that CBD not only inhibits biofilm formation by *C. albicans* SC5314 but can disperse extant biofilms [46]. Of late, low efficacy anti-*Cryptococcus neoformans* ATCC 90113 activity of ergost-5-en-3-ol from cannabis roots from a high CBD variety has also been reported [26]. Hemp extracts have likewise been reported to exhibit anti-fungal activity against the tinea pedis pathogens, *Trichophyton interdigitale* and *Trichophyton rubrum* [47].

On the other hand, Tazi et al. found that cannabis smoke condensate (CSC), produced from commercial cigarettes made from an unspecified *C. sativa* variety, was able to enhance *C. albicans* ATCC SC5314 biofilm mass and to increase hyphal length, both likely to contribute to *C. albicans* pathogenesis [64].

It is hoped that, as with the recent renaissance in the identification of cannabis-informed anti-bacterial agents, further insight into prospective anti-mycotic activities might be gained in the coming years.

### 2.4. Anti-Viral Activities of Cannabis, Cannabis-Derived Compounds

McDew-White et al. have noted that the long-term, low dose administration of THC inhibits proinflammatory gene expression and alters infection-related miRNA profiles in the gingiva, as well as the relative salivary abundance of several groups of bacteria, in chronically SIV-infected macaques, relative to non-THC treated animals [65]. These THC-mediated events are at least partly CB2-related and protective against SIV-enhanced periodontal disease. DeMarino et al. have reported that CBD reduces extracellular vesicle release from HIV-infected monocytic cells and their viral cargo [66]. Otherwise, recent studies of the anti-viral properties of CIMPs are limited in number and are SARS-CoV-2 centric, as summarized in Table 5.

There is evidence, albeit limited, that cannabidiol (CBD) may have potential as an anti-SARS-CoV-2 agent [48,67,68,69,70,71] presented concomitantly with important warnings against prescription of cannabinoid products for COVID-related symptoms at present [50,72]. For example, CBD has been suggested to be an efficient inhibitor of SARS-CoV-2 (strain 229E) replication in human lung fibroblasts (MRC-5) through enhancement of antiviral terpene efficacy [68]. Both THC and CBD have been reported to interact with the Mpro protein of SARS-CoV-2 and to exhibit anti- SARS-CoV-2 activities with an IC50 of 10.25 μM and 7.91 μM, respectively [48]. Moreover, Esposito et al. [69] have hypothesized that CBD may have some adjunctive anti-COVID efficacy, as CBD administration can down-regulate the expression of SARS-CoV2 receptors (ACE2, transmembrane proteinase 2 [TMPRSS2]) in 3D human epithelial tissue models, as initially reported by Wang et al. [70].

Van Breemen et al. report that CBDA and CBGA interact with the SARS-CoV-2 spike protein S1 subunit (Kd = 5.6 ± 2.2 μM and 19.8 ± 2.7 μM, respectively) and prevent the entry of several live viral variants into human epithelial cells (see Table 5) [49]. The authors conclude that a combination of CBDA and CBGA may represent a greater adjunctive challenge to SARS-CoV-2 spread. Recent reviews of the cannabis-related, SARS-CoV-2-specific literature, which accentuate the need for further research including clinical trials, are available [15,73,74].

Thus, while the development of cannabis-informed therapeutic strategies to treat viral infections are theoretically possible, this is a generally underdeveloped research area.

### 2.5. Antimicrobials as Therapeutics for Neurological Disorders

It must be recognized that a potential microbial etiology for neurodegenerative diseases has been postulated for some time, with the oral and gut microbiota particularly implicated, as reviewed extensively elsewhere [75,76,77,78,79,80,81,82,83,84]. This has fostered the possibility of the development of suitable bacteria-targeted therapeutic strategies, some of which show promise in animal models [80,81,84,85,86]. It remains to be clarified if it is a general dysbiosis, specific bacteria and their virulence factors, or both that may be most important in the development of Parkinson’s disease, Alzheimer’s disease and other neuropathologies. In addition to direct mechanisms, etiological roles for bacterial-induced inflammation and an irregular microbial metabolome have also been posited [75,76,78,79,80]. Therefore, the unintended consequences of CB2-agonists with antimicrobial properties may be hard to predict. On the one hand, chronic administration of an anti-bacterial agent could induce a systemic dysbiosis or promote the emergence of neurodegenerative disease-associated species, such as phytocannabinoid-resistant *Treponema denticola* or other spirochetes [32,87,88], that could promote disease. On the other, the suppression of specific neurodegenerative disease-associated bacteria by CB2 agonists, such as the phytocannabinoid-responsive pathogens *Porphyromonas gingivalis* or *Helicobacter pylori* [32,82,83,87,89,90], may represent a relevant, novel therapeutic approach. In any case, antimicrobial properties (e.g., Table 1, Table 2, Table 3, Table 4 and Table 5) should be considered during the selection and development of potential neuroprotective CB2-engaging biomolecules.

## 3. Translational Considerations for the Development of Cannabis-Informed Antimicrobials

### 3.1. In Vivo and Ex Vivo Verification of Antimicrobial Activities

One of the more promising translational opportunities for phytocannabinoids, in the context of infectious diseases, may be the incorporation of phytocannabinoids into oral rinses. Vasudevan and Stahl have noted the efficient killing of aerobic plaque bacteria by CBD- and CBG-infused (1% by weight) mouthwashes [91]. Interestingly, the preparations were alcohol-free and exhibited similar efficacy to 0.2% chlorhexidine. The same research group has suggested that the incorporation of CBD into dental polish can assist in plaque removal [92]. On the other hand, also in the oral context, Gu et al. have shown that cannabinoids, including CBD, exhibit differential activities against common bacterial components of dental plaque. For example, *Treponema denticola* appears phytocannabinoid-resistant while *Filifactor alocis* is sensitive. The authors have suggested that, should such a phenomenon occur in vivo, phytocannabinoids may help promote the oral dysbiosis that is the hallmark of periodontitis [32], a condition seemingly enhanced in cannabis smokers, as reviewed recently [87].

CBD, depending on the formulation of the delivery vehicle, has been shown to be efficient at killing *S. aureus* on porcine skin, suggesting topical delivery systems may be a useful approach [11]. In vivo verification of the antimicrobial activities of CIMPs, however, is another area much in need of research attention. One particularly important obstacle to their in vivo utility may be the potential for serum binding and inactivation of phytocannabinoids, as reported for CBD, which would clearly limit systemic applications [8,11]. However, Farha et al. have shown that a single CBG bolus, delivered i.p., exhibited efficient antibacterial activity in a murine model of *S. aureus* infection, in which the splenic bacterial burden was monitored [12]. The comprehensive study of the anti-microbial properties of CBG by Farha et al. did note that this cannabinoid induced erythrocyte lysis, albeit at 16-fold the MIC, raising another concern about systemic use [12]. However, such toxicological complications have been rarely noted in the extant literature.

Addiction-related factors as well physiological issues, particularly developmental, arising from exocannabinoid engagement of endocannabinoid receptors, must also be considered, as has been discussed by Farha et al. [12]. The potential for immune suppression [32] is another possible area of concern. However, it must be noted that most antibacterial regimens are intended to be of acute, rather than chronic, duration. This review of the literature reinforces that multiple aspects of the use of CIMPs remain to be elucidated in detail.

### 3.2. Optimization of the Anti-Microbial Potential of Cannabis-Derived Compounds

There are a number of factors related to the optimization or, for that matter, negation of cannabis-based anti-microbial activities to be considered. Upon extracting and characterizing *C. sativa* essential oils, Palmieri et al. established a wide range of essential oil components identified from different cultivars and with differing extraction methods, coincident with a range in antimicrobial capacities [34]. Of 5 varieties tested the highest antimicrobial performance was observed for *C. sativa* GSK [34]. This, and other studies, suggest that antimicrobial components found in cannabis plants can be optimized horticulturally, as well as technologically. It can be imagined that non- or minimally-psychotropic molecules or compounds, as summarized and differentiated by Klahn [14], could be prioritized over psychotropic agents for both cognitive and antimicrobial applications.

In a search for novel compounds with antimicrobial activities in cannabis plant roots, an understudied area, Elhendawy et al. reported that *p*-coumaroyltyramine demonstrates potent activity against *E. coli* [26]. Interestingly, *p*-coumaroyltyramine has previously been isolated from *Glycosmis pentaphylla* (orangeberry; gin berry) and shown to have photo-activated antimicrobial activity against the Gram-positive bacteria, *S. aureus* and *Bacillus subtilis* [93]. *Capsicum annuum* infection with the black rot bacterium, *Xanthomonas campestris*, has been reported to result in *p*-coumaroyltyramine accumulation [94]. Similarly, this compound is upregulated upon *Pseudomonas syringae* infection of tomato plants (*Solanum lycopersicum*) with the authors suggesting that *p*-coumaroyltyramine could be involved in the antibacterial defenses of plants [95]. This hypothesis is supported by data showing that the classical TLR4 agonist, LPS from Gram negative bacteria, altered the kinetics of *p*-coumaroyltyramine induction upon subsequent infection with *Xanthamonas* spp., again in *C. annuum* [96]. Of three *C. sativa* varieties tested, *p*-coumaroyltyramine content was greatest in a high CBD variety [26], further emphasizing the potential for the development and cultivation of varieties with specific desired antimicrobial properties. Jin and Lee examined hexane extracts of hemp seeds and showed anti-*Propionibacterium acnes* efficacy aligned with a suppressed innate response to infection in human HaCaT keratinocytic cells [43]. Thus, while the leaves and flowers have received the most attention, investigation of other parts of the cannabis plant may also prove useful.

Technologies that will improve traditional extraction yields are under development [8] but beyond the scope of the present review. Further, there is the potential to optimize CIMPs by improving delivery systems or via the development of stable, chemically altered, synthetic or mimetic molecules, akin to the antiemetic THC analogue, Nabilone. For example, Blaskovich et al. have reported that differing CBD formulations (silicones, mineral oil jelly; diethylene glycol monoethyl ether and polyethylene glycol) exhibit wide variations in anti-*S. aureus* efficacy in a cutaneous infection system [11].

**Figure 1:**
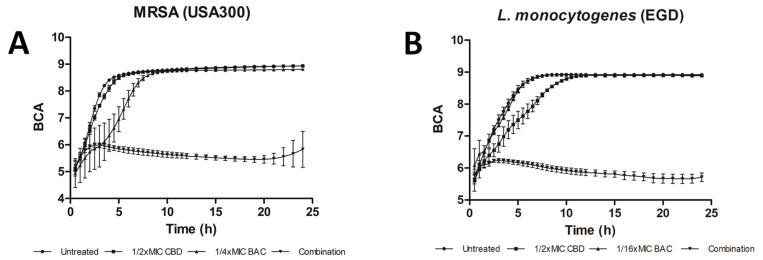
**CBD enhances antibacterial efficacy of bacitracin against Gram-positive bacteria.** Growth curves of cannabidiol (CBD) in combination with bacitracin (BAC). The bacterial density of (**A**) Methicillin-resistant *Staphylococcus aureus* USA300 FPR 3757 and (**B**) *Listeria monocytogenes* EGD was monitored over 24 h. Other investigators have reported that CBD alone is active against certain *S. aureus* and *L. monocytogenes* strains, as summarized in Table 2. BCA: Background corrected absorption. Full details provided in the primary manuscript. (Figure 1 is reproduced from Wassman et al. [41], which was published under the Creative Commons Attribution 4.0 (CC BY 4.0) International License.).

The use of cannabinoids as antimicrobial adjuncts, rather than as standalone therapeutics, has also been considered. For example, the combination of polymyxin B and CBG is efficient at killing *E. coli* [12], and CBD seems to enhance the efficacy of polymyxin B against multiple strains of *A. baumanni*, *K. pneumoniae* and *P. aeruginosa* [31,97]. The independent and combined efficacy of CBD and bacitracin against *S. aureus* and *L. monocytogenes* is presented in Figure 1, as extracted from Wassman et al. [41]. Antezana et al. noted that silver nanoparticles loaded collagen hydrogels enhanced with *C. sativa* oil extract exhibited prolonged (7 day) and efficacious anti-microbial activity against both Gram-positive (*S. aureus* ATCC 29213) and Gram-negative (*P. aeruginosa* ATCC 27853) bacteria, with the cannabis oil substantially decreasing epithelial cell cytotoxicity [98]. Kosgodage et al. have reported that CBD enhanced the bactericidal action of selected antibiotics against Gram-negative bacteria [99]. Thus, the use of CIMPs to augment available therapeutics is another avenue by which their utility may be improved.

Critically, optimization and negation—as may be attractive for neuroprotective applications—of cannabis-based anti-microbial agents is likely to be greatly facilitated by an improvement in our knowledge of their mechanisms of action, and consideration of the characteristics of the target microbe [11], not least at the ultrastructural level.

### 3.3. Anti-Microbial Mechanisms

Efficacious cannabinoid receptor agonism accompanied by minimal antimicrobial activity would be attractive properties of CB2-directed neuroprotective mediators. Conversely, minimal CB2-engagement and maximal antimicrobial efficiency would be attractive properties for novel CIMP-based agents to be used for the treatment of infectious diseases. The anti-microbial mechanisms of CIMPs, then, need to be better understood.

**Figure 2:**
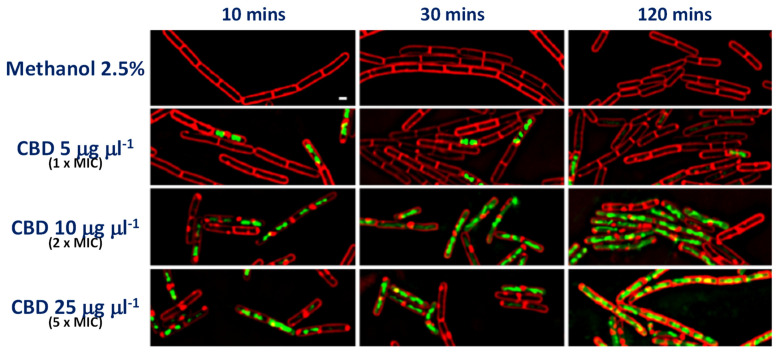
**CBD induces membrane permeability in Gram-positive bacteria.** Bacterial cytological profiling (BCP) assay in *B. subtilis* PY79 showing uptake of SYTOX™ Green dye over time in the presence of increased concentrations of CBD. Red FM 4–64 dye is used to visualize membranes. The white scale bar represents 1 µm. The authors also established that the same phenomenon occurs in *S. aureus* ATCC 29213. (Figure 2 is reproduced from Blaskovich et al. [11], which was published under the Creative Commons Attribution 4.0 (CC BY 4.0) International License).

Anti-bacterial mechanisms are, perhaps, best studied in *S. aureus*. Protein, DNA, RNA and peptidoglycan production are all suggested to be shut down by CBD, as determined in radiolabeled macromolecular synthesis assays [11]. Lipid synthesis was curtailed at sub-MIC concentrations of CBD with rapid membrane polarization apparent, as visualized by SYTOX™ Green dye uptake by coccoidal (*S. aureus*) and rod-shaped (*B. subtilis*) Gram-positive bacteria [11], as presented in Figure 2.

The mechanism(s) of action of CBG against the caries-associated pathogen, *S. mutans*, has been associated with membrane hyperpolarization and decreased membrane fluidity, septal invagination and altered metabolic activity in strain ATCC 700610 [45]. Wassman et al. reported altered septum formation and suppression of the gene activity of the key cell division regulator, *ezrA*, in CBD and bacitracin-treated *S. aureus* (USA300 FPR3757) [41]. Similarly, Farha et al. observed that CBG appears to target the cytoplasmic membrane of *S. aureus* [12]. Aqawi et al. noted that CBD inhibition of *S. mutans* growth is accompanied by the suppression of genes involved in extracellular polysaccharide synthesis (*gftB*, *gftC*, *gftD*, *ftf*), quorum sensing (*luxS*, *ComD*, *ComE*), biofilm formation, including *gbpA*, *gpbB*, *spaP*, *vicR*, *wapA*, and *brpA*, acid tolerance (*relA*, *atbB*) and stress responses (*sod*, *nox*, *groEL*, *dnaK*). Importantly, the authors also report that bacterial cell division is not requisite for CBG efficacy [57]. Interestingly, Marini et al. observed the influence of essential oils from hemp on *L. monocytogenes* virulence traits. They noted significantly suppressed *prfA*, *flaA*, *motA* and *motB* gene activity, accompanied by fewer flagella and reduced motility; and reduced epithelial cell entry capacity in the majority (7/8) of the invasive strains tested, compared to control bacteria [42].

Feldman et al. have reported that CBD not only inhibits biofilm formation by *C. albicans* SC5314 but can also disperse extant biofilms. The mechanism of action may be both multifactorial and different to established anti-fungal agents. CBD perhaps acts through the downregulation of exopolysaccharide synthesis, induction of mitochondrial membrane hyperpolarization, altering plasma membrane permeability, modifying the fungal cell wall chitin content and promoting yeast- over hyphal-associated gene expression [46].

As for potential mechanisms of action of cannabinoids as anti-viral agents, the most recent evidence comes from the SARS-CoV-2 pandemic. CB1 and CB2, at least at the mRNA level, are upregulated in circulating immune cells in individuals with moderate to severe disease [100]. One report found that several cannabinoids, including tetrahydrocannabivarin (THCV), THC, CBG, and CBN, are computationally predicted to interact with mRNAs encoding proteins assumed to be involved in SARS-CoV-2 replication, translation, assembly and release (ORF1ab, surface glycoprotein, envelope protein, nucleocapsid phosphoprotein) [101], although effective inhibition of such a broad range of translational events has yet to be confirmed in vitro or in vivo. Altyar et al. screened, in silico, 45 cannabinoids for their potential to interact with key SARS-CoV-2 enzymes, noting that cannabichromanon (CBCN) was best suited as a potential inhibitor of MPro and Plpro [102]. As noted earlier, Breemen et al. reported that CBGA, CBDA and tetrahydrocannabinolic acid (THCA) interact with the SARS-CoV-2 S1 spike protein subunit [49]. Nguyen et al. have evidenced that CBD and 7-OH-CBD, but not others in a bank of cannabinoids tested (CBG, CBC, CBDA, CBDV, THC), inhibit SARS-CoV-2 replication in epithelial cells in a manner associated with inhibition of viral genes, including almost complete shutdown of the activity of those encoding the spike protein and nucleocapsid, reversion of viral influence on host genes and upregulation of the IFN anti-viral axis [50]. Intriguingly, the authors also report that CBD consumption by humans correlates with a highly significant reduction in SARS-CoV-2 relative to control subjects [50]. Most recently, Fernandes et al. have reported that CBD enhances the anti-viral response of SARS-CoV-2-infected epithelial cells, but not uninfected cells, as determined by activation of interferon and 2′-5′-oligoadenylate synthetase (OAS) genes [103].

**Figure 3:**
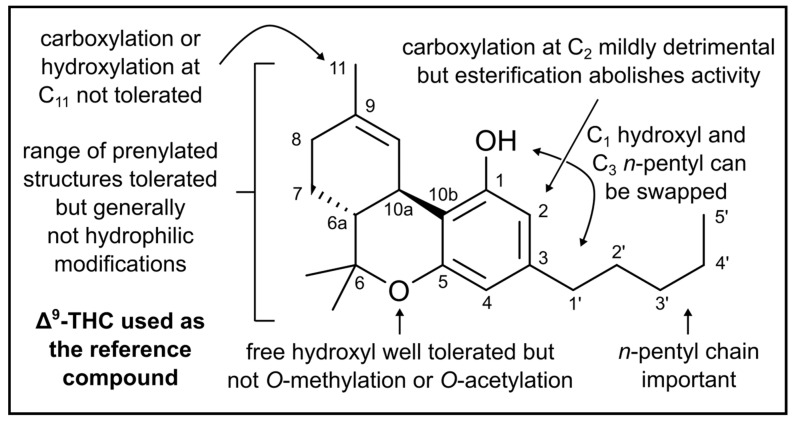
**Summary of cannabinoid structure-antimicrobial activity relationships in the context of Gram-positive bacteria.** Figure 3 is reproduced from Karas et al. [8], which was published under the Creative Commons Attribution 4.0 (CC BY 4.0) International License.

Clearly, structure-function analyses will be key in the development of cannabis-educated antimicrobial and non-antimicrobial therapeutics. To this end, Karas et al. [8] have reviewed the available evidence on cannabinoids variants highlighting the key molecular features known to control anti-Gram-positive activities, as summarized in Figure 3. Elements proffering effective CB2 agonism in CIMPS require further elucidation, as discussed below, while, and at the same time, there is need to better understand CIMP-mediated bactericidal and bacteriostatic mechanisms of action.

## 4. Translational Considerations for the Development of Non-Antimicrobial CB2-Engaging CIMPs

Above, we have commented on attempts to design CIMPs with improved antimicrobial potencies for the control of infectious diseases. However, with respect to the use of CB2 agonists as therapeutics for variant neuropathologies, the opposite approach would seem appropriate. This is highlighted by the growing evidence suggesting an association between the flux in the gut microbiome and neurological disorders such as Alzheimer’s and Parkinson’s diseases, with contemporary reviews available [79,104,105]. Osman et al. have shown that photooxygenation of some cannabinoids resulted in multiple derivates, some with enhanced CB2 binding affinity, some with altered antimicrobial profiles [39]. Clearly the combination of both—higher receptor affinity with lower microbiocidal activity—may be of the most interest for neurological translation. Another recent report states that while CBCA itself is more efficient than vancomycin in killing against *E. coli* and *S. aureus*, several derivative synthetics (cannabichromene methyl ester trifluoroacetate [CBCTFA], cannabicyclol methyl ester [CBLM], cannabichromene methyl ester [CBCM], cannabidivarin methyl ester [CBDVM]) are not [55]. Equally, Karas et al. elucidate multiple cannabinoid variants in which antimicrobial activities are reduced or abolished [8]. Establishment of their interactions with CB2, then, will be enlightening. Figure 3 presents the structural alterations known to reduce or abolish cannabinoid activity against Gram-positive bacteria, using THC as the model molecule.

Meantime, negative data from existent CIMP–microbe interaction studies are likely to be particularly enlightening. It is the nature of modern science, however, that such data are not prioritized for publication although insights are available. As for bacteria, certain cannabis-derived CB2-agonists are inactive or poorly active against individual strains of *Acinetobacter baumannii*, *Serratia marcescens*, *Stenotrophomonas maltophila*, *Burkholderia cepacia*, *Proteus mirabilis* and *Shigella dysenteriae*, all Gram-negatives [11]. Similarly, Klahn has summarized the great variation in antimicrobial activities of a wide bank of understudied phytocannabinoids and derivatives against a panel of bacteria and fungi. Several of the identified compounds show promise as having low to no antimicrobial activity [14]. It is hoped that this review article may spark further such interest in the identification or development of microbiologically neutral CB2 agonists.

## 5. Emergence of Microbial Resistance to Cannabis and Cannabis-Derived Compounds

The emergence of multiple resistance traits is the cause of the critical reduction in the efficacy of available antibiotics. The use of non-medicinal cannabis and medical CB2 agonists, including phytocannabinoids, to treat neurological and other disorders has the potential to assist in the development of CIMP-resistant microbes. Interestingly, however, several studies have suggested that there is a low predisposition for emergent bacterial resistance to CIMPs among those bacteria examined. These include *S. aureus*, as assessed by innate resistance frequency and multi-passaging, with similar data generated in *Cutibacterium acnes* [11]. Farha et al. similarly noted that no spontaneous resistant mutants of *S. aureus* emerged after multiple passages at supra-MIC cannabinoid (CBG) concentrations [12]. It is important to note that, while resistance can always develop through multiple mechanisms, it appears that bacterial biotransformation of a key antimicrobial cannabinoid, CBD, may be infrequent [106]. Additionally, CBD has been reported to suppress the release of membrane vesicles from several bacterial species, a phenomenon associated with resistance transfer [99]. Further, in the most extensive related study to date, physiological alteration of CBD was found to be a rare feature among a panel of microbes, primarily fungi (*Mucor ramannianus*, *Beauveria bassiana* and *Absidia glauca*) [106]. Again, the potential for resistance to phytocannabinoids and other cannabis-derived chemicals, needs to be further investigated. Studies of resistance mechanisms in naturally resistant microbes, such as *Treponema denticola*, would appear reasonable, alongside attempts to induce resistance in sensitive microorganisms. Interestingly, Wassman and colleagues have recently suggested that, while other CBD resistance traits were noted, mutations in genes encoding an *S. aureus* efflux pump, *farE* (a CBD-induced gene), and/or its regulator, *farR*, are present in all CBD-resistant strains examined. While recreation of specific *farER* system mutants did not bestow CBD resistance, it did confer reduced susceptibility to bacitracin/CBD combinations [107].

## 6. Discussion

In addition to contemporary summations of the immunoregulatory [13] and antimicrobial properties of cannabinoids [8,14,15], including an excellent overview of the older literature describing MICs of cannabinoids [14], others addressing emerging extraction technologies [8], cannabinoid structures and their relation to activity [14], cannabinoid abundances [14], their role in variant diseases [108] and psychotropic activities [14], as well as CB2 receptors themselves, are also available. The summary of evidence suggesting that CBG may represent a lead antimicrobial cannabinoid is illuminating [12]. Further, there is a pressing interest in the potential of cannabinoids, such as CBD, to inhibit SARS-CoV-2 cellular entry and replication through varying mechanisms [48,49,101]. This review augments these prior articles by focusing on the most recent literature on cannabis-related antimicrobials, primarily summarized in Table 1, Table 2, Table 3, Table 4 and Table 5, and providing a synopsis of the knowledge gaps that act as a barrier to the acceptance and utility of cannabinoid-based antimicrobial therapeutics.

While the potential of cannabis to represent a source of novel antimicrobials is clear, such key knowledge gaps include (i) the cellular and molecular characteristics of sensitive versus resistant bacteria; (ii) efficient delivery vehicles, particularly for systemic applications; (iii) a more complete understanding of antimicrobial mechanisms; (iv) screening of understudied minor *C. sativa*-derived components; (v) the development of improved efficiency synthetics and mimetics; (vi) the use of phytocannabinoids, and other cannabis-derived compounds, as adjuncts to established antibiotics; and (vii) the potential for unwanted side-effects, including immune suppression and the consequences of endo- and phytocannabinoid interactions. However, with the resurgence in interest in CB2 agonists and CIMPs, in general, as antimicrobials, much research progress can be expected in the coming years. It must be hoped that clinical applications can soon be developed and tested in order to answer more definitively if the multitudinous antimicrobial properties ascribed to CIMPs are truly translatable. In the meantime, with no accepted clinical uses for phytocannabinoids or other cannabis-related entities in the context of infectious diseases, the antimicrobial promise of CIMPS remains as yet unrealized potential.

From a neuroprotective viewpoint, molecules that engage the CB2 receptor show promise as therapeutic agents for neuroinflammatory and neurodegenerative diseases—the focus of this Special Issue. However, some CIMPs have been reported to act as potent antimicrobial agents. The microbicidal properties ascribed to several CB2 receptors should be considered when designing therapeutic approaches for neurocognitive disorders, particularly in the case of long-term strategies. Finally, identification and development of neuroactive agents that are efficient CB2 agonists, but non-antimicrobial may represent an attractive strategy.

## Figures and Tables

**Table 1:** Efficacy of cannabis-derived molecules and preparations against Gram-negative bacteria.

Compound	Source	Target	Strain(s)	Efficacy	Reference
*p*-coumaroyltyramine	Roots of *C. sativa*, high CBD variety	*Escherichia coli*	ATCC 35218	0.8 μg/mL *	Elhendawy et al., 2019 [26]
Water extract	*C. sativa,* Futura 75	*Escherichia coli*	ATCC 10536	7.1 mg/mL **	Ferrante et al., 2019 [27]
Hemp stem Ag-nanoparticles	*C. sativa,* USO-31	*Escherichia coli*	UTI 89	12.5 µg/mL ** 25 µg/mL ***	Singh et al., 2018 [28]
CBD	Commercial	*Escherichia coli*	ATCC 13762	29 µM *	Russo et al., 2021 [29]
Cannabidivarin (CBDV)	Commercial	*Escherichia coli*	ATCC 13762	35 µM *	Russo et al., 2021 [29]
Essential oils	*C. sativa* Futura 75	*Helicobacter pylori*	14 strains, variant Ab sensitivity patterns	8–64 µg/mL ** 8–64 µg/mL ***	Zengin et al., 2018 [30]
Cannabidiol (CBD)	Commercial	*Legionella pneumophila*	MMX 7515	1 µg/mL **	Blaskovich et al., 2021 [11]
CBD	Commercial	*Moraxella catarrhalis*	MMX 3782	1 µg/mL **	Blaskovich et al., 2021 [11]
CBD	Commercial	*Moraxella catarrhalis*	ATCC 25238	164 µg/mL **	Abichabki et al., 2022 [31]
CBD	Commercial	*Neisseria gonorrhoeae*	ATCC 49226	1 µg/mL **	Blaskovich et al., 2021 [11]
CBD	Commercial	*Neisseria meningitidis*	ATCC 13090	0.25 µg/mL **	Blaskovich et al., 2021 [11]
CBD	Commercial	*Neisseria meningitidis*	ATCC 13077	128 µg/mL **	Abichabki et al., 2022 [31]
CBD	Commercial	*Porphyromonas gingivalis*	ATCC 33277	5 µg/mL *	Gu et al., 2019 [32]
Water extract	*C. sativa*, Futura 75	*Pseudomonas aeruginosa*	ATCC 15442	7.1 mg/mL **	Ferrante et, 2019 al [27]
Hemp stem Ag-nanoparticles	*C. sativa,* USO-31	*Pseudomonas aeruginosa*	PAO1	6.25 µg/mL ** 12.5 µg/mL ***	Singh et al., 2018 [28]
Cannabinol oil extract	Commercial	*Pseudomonas aeruginosa*	ATCC 9027	2% **	Di Onofrio et al., 2019 [33]
Essential oils	*C. sativa* (Futura 75, Carmagnola Lemon, Gran Sasso Kush, Carmagnola, Kompolti)	*Pseudomonas fluorescens*	P34	0.3–2.5 µL/mL **	Palmieri et al., 2021 [34]

* IC50; ** MIC (Minimum inhibitory concentration); *** MBC (Minimum bactericidal concentration).

**Table 2:** Efficacy of cannabis-derived molecules and preparations against *Staphylococcus aureus*.

Compound	Source	Strain(s)	Antibiotic Sensitivity	Efficacy	Reference
Hexane extracts	*C. sativa*, Fibrante	19 clinical strains	All MRSA	4.9–78.1 μg/mL ***	Muscara et al., 2021 [35]
Hexane extracts	*C. sativa*, Fibrante	ATCC 6538	Methicillin-sensitive	4.9 μg/mL ** 4.9–19.5 ***	Muscara et al., 2021 [35]
Hexane extracts	*C. sativa*, C-309	19 clinical strains	All MRSA	39.1–78.1 μg/mL ***	Muscara et al., 2021 [36]
Hexane extracts	*C. sativa*, C-309	ATCC 6538	Methicillin- sensitive	39.1 μg/mL ** 39.1–78.1μg/mL ***	Muscara et al., 2021 [36]
CBD	Purified from *Cannabis sativa*, fiber types	USA300	MRSA	1 μg/mL **	Martinenghi et al., 2020 [37]
CBD	Purified from *Cannabis sativa*, fiber types	ATCC 25923	Methicillin- sensitive	1 μg/mL **	Martinenghi et al., 2020 [37]
Cannabidiolic acid (CBDA)	Purified from *Cannabis sativa*, fiber types	USA300	MRSA	4 μg/mL **	Martinenghi et al., 2020 [37]
CBDA	Purified from *Cannabis sativa*, fiber types	ATCC 25923	Methicillin- sensitive	2 μg/mL **	Martinenghi et al., 2020 [37]
Essential oil	*C. sativa*, Futura 75	STA 32, St 47, St 39		1.25–5 μL/mL ** 1.25–5 μL/mL ***	Pellegrini et al., 2020 [38]
Essential oils	*C. sativa* (Futura 75, Carmagnola Lemon, Gran Sasso Kush, Carmagnola, Kompolti)	STA 32, St 47		0.156–20 µL/mL **	Palmieri et al., 2021 [34]
Water extract	*C. sativa* L., Futura 75	ATCC 6538s	Disinfectant testing strain	3.6 mg/mL **	Ferrante et al. 2019 [27]
Essential oils	*C. sativa* L., Futura 75	ATCC 29213, 101TV, 104, 105	Variant Ab sensitivity patterns	8 mg/mL ** 16 mg/mL *** 16–24 mg/mL ****	Zengin et al., 2018 [30]
Oxygenated derivatives of Δ9-THC and its isomer Δ8-THC	*-*	Not presented	Not noted	2.5–5 μg/mL *	Galal Osman et al., 2018 [39]
Oxygenated derivatives of Δ9-THC and its isomer Δ8-THC	*-*	Not presented	MRSA	2.5–10 μg/mL **	Galal Osman et al., 2018 [39]
CBD	Commercial	ATCC 25923, ATCC 43300, NRS-1, VRS1	MMSA, MRSA, MRSA (vancomycin intermediate) and VRSA, respectively	1–4 µg/mL **	Blaskovich et al., 2021 [11]
Cannabigerol (CBG)	Lab synthesized	USA300	MRSA	4 µg/mL ****	Farha et al., 2020 [12]
Various essential oils	Multiple sources	ATCC 6538, 18As, 386	Ciprofloxacin sensitive	2–32 µg/mL **	Iseppi et al., 2019 [40]
Various terpenes	Commercial	ATCC 6538, 18As, 386	Ciprofloxacin sensitive	4–32 µg/mL **	Iseppi et al., 2019 [40]
CBD	Commercial	ATCC 6538, 18As, 386	Ciprofloxacin sensitive	8–32 µg/mL **	Iseppi et al., 2019 [40]
CBD	Commercial	MRSA USA300	MRSA	4 µg/mL **	Wassman et al., 2020 [41]
CBD	Commercial	ATCC 6538	Ciprofloxacin sensitive	1.8 µM *	Russo et al., 2021 [29]
CBDV	Commercial	ATCC 6538	Ciprofloxacin sensitive	30.1 µM *	Russo et al., 2021 [29]
CBD	Commercial	ATCC 29213, ATCC 43300, N315, ATCC 700698, ATCC 700699, ATCC BAA-976, ATCC BAA-977	Variant Ab susceptibility patterns	4 µg/mL**	Abichabki et al., 2022 [31]

* IC50; ** MIC (minimum inhibitory concentration); *** MBC (minimum bactericidal concentration); **** MBEC (minimum biofilm eradication concentration).

**Table 3:** Efficacy of cannabis-derived molecules and preparations against other Gram-positive bacteria.

Compound	Source	Target	Strain(s)	Efficacy	Reference
Essential oils	Various sources	*Bacillus isolates*	*n* = 12, including *B. cereus*	0.5–32 µg/mL **	Iseppi et al., 2019 [40]
Various terpenes	Commercial	*Bacillus isolates*	*n* = 12, including *B. cereus*	1–32 µg/mL **	Iseppi et al., 2019 [40]
CBD	Commercial	*Bacillus isolates*	*n* = 12, including *B. cereus*	2–16 µg/mL **	Iseppi et al., 2019 [40]
Essential oils	*C. sativa* (Futura 75, Carmagnola Lemon, Gran Sasso Kush, Carmagnola, Kompolti)	*Brochothrix thermosphacta*	B1	0.31–20 µg/mL **	Palmieri et al., 2021 [34]
CBD	Commercial	*Cutibacterium acnes*	ATCC 6919	1–2 µg/mL **	Blaskovich et al., 2021 [11]
CBD	Commercial	*Clostridioides difficile*	M7404	2–4 µg/mL **	Blaskovich et al., 2021 [11]
CBD	Commercial	*Enterococcus casseliflavus*	ATCC 12361	4 µg/mL **	Abichabki et al., 2022 [31]
CBD	Commercial	*Enterococcus faecium*	ATCC 35667, ATCC 700221, ATCC 19434, MMX 485	0.5–1 µg/mL **	Blaskovich et al., 2021 [11]
Essential oils	Various sources	*Enterococcus faecium*	V5, EQ19	1–32 µg/mL **	Iseppi et al., 2019 [40]
Various terpenes	Commercial	*Enterococcus faecium*	V5, EQ19	1–16 µg/mL **	Iseppi et al., 2019 [40]
CBD	Commercial	*Enterococcus faecium*	V5, EQ19	1–4 µg/mL **	Iseppi et al., 2019 [40]
Essential oils	*C. sativa* (Futura 75, Carmagnola Lemon, Gran Sasso Kush, Carmagnola, Kompolti)	*Enterococcus faecium*	ATCC 19434	1.25->20 µg/mL **	Palmieri et al., 2021 [34]
CBD	Commercial	*Enterococcus faecalis*	NCTC 7171, ATCC 51559,ATCC 29212, ATCC 51299	2–4 µg/mL **	Abichabki et al., 2022 [31]
CBD	Commercial	*Enterococcus faecalis*	ATCC 29212, clinical isolate, MMX 486	1–4 µg/mL **	Blaskovich et al., 2021 [11]
CBD	Commercial	*Enterococcus faecalis*	13-327129	8 µg/ml	Wassman et al., 2020 [41]
Essential oils	Various sources	*Enterococcus faecalis*	ATCC 29212, V3, V4, v6	0.5–32 µg/mL **	Iseppi et al., 2019 [40]
Various terpenes	Commercial	*Enterococcus faecalis*	ATCC 29212, V3, V4, v6	0.5–16 µg/mL **	Iseppi et al., 2019 [40]
CBD	Commercial	*Enterococcus faecalis*	ATCC 29212, V3, V4, v6	1–4 µg/mL **	Iseppi et al., 2019 [40]
CBD	Commercial	*Enterococcus gallinarum*	ATCC 12359	4 µg/mL **	Abichabki et al., 2022 [31]
Essential oils	Various sources	*Enterococcus hirae*	ATCC 10541	2–32 µg/mL **	Iseppi et al., 2019 [40]
Various terpenes	Commercial	*Enterococcus hirae*	ATCC 10541	1–8 µg/mL **	Iseppi et al., 2019 [40]
CBD	Commercial	*Enterococcus hirae*	ATCC 10541	2 µg/mL **	Iseppi et al., 2019 [40]
CBD	Commercial	*Filifactor alocis*	ATCC 35896	1 µg/mL *	Gu et al., 2019 [32]
Essential oil-derived α-pinene and myrcene	*C. sativa*, Futura 75	*Listeria monocytogenes*	11 clinical isolates	≥1024 µg/mL ***	Marini et al. 2018 [42]
CBD	Commercial	*Listeria monocytogenes*	EGD	4 µg/ml	Wassman et al., 2020 [41]
Essential oils	*C. sativa* (Futura 75, Carmagnola Lemon, Gran Sasso Kush, Carmagnola, Kompolti)	*Listeria monocytogenes*	ATCC 7644, ATCC 19114, LM4	0.6->20 µL/mL **	Palmieri et al., 2021 [34]
Essential oils	Various sources	*Listeria monocytogenes*	NCTC 10888, ATCC 13932, ATCC 5008, 70, 139	2–32 µg/mL **	Iseppi et al., 2019 [40]
Various terpenes	Commercial	*Listeria monocytogenes*	NCTC 10888, ATCC 13932, ATCC 5008, 70, 139	0.5–4 µg/mL **	Iseppi et al., 2019 [40]
CBD	Commercial	*Listeria monocytogenes*	NCTC 10888, ATCC 13932, ATCC 5008, 70, 139	1–4 µg/mL **	Iseppi et al., 2019 [40]
Essential oil	*C. sativa* L, Futura 75	*Listeria monocytogenes*	ATCC 19114, LM 4, ATCC 7644	2.5–5 μL/mL ** 2.5–5 μL/mL ***	Pellegrini et al., 2020 [38]
CBD	Commercial	*Micrococcus luteus*	CCT 2688	4 µg/mL **	Abichabki et al., 2022 [31]
Hexane extract	*C. sativa* (unspecified hemp variety) seeds	*Propionibacterium acnes*	KCTC strain	20% extract **	Jin et al., 2018 [43]
CBD	Commercial	*Rhodococcus equi*	ATCC 6939	4 µg/mL **	Abichabki et al., 2022 [31]
CBD	*C. sativa* extraction	*Salmonella newington*	UC1698	0.125 µg/mL **	Gildea et al., 2022 [44]
CBD	*C. sativa* extraction	*Salmonella typhimurium*	MS1868	0.125 µg/mL **	Gildea et al., 2022 [44]
CBD	Commercial	*Staphylococcus agalactiae*	ATCC 13813	4 µg/mL **	Abichabki et al., 2022 [31]
CBD	Commercial	*Staphylococcus epidermidis*	933010 3F-16 b4	4 µg/ml	Wassman et al., 2020 [41]
CBDA	Purified from *Cannabis sativa*, fiber types	*Staphylococcus epidermidis*	CA#71, ATCC 51625	4 μg/mL **	Martinenghi et al., 2020 [37]
CBD	Purified from *Cannabis sativa*, fiber types	*Staphylococcus epidermidis*	CA#71, ATCC 51625	2 μg/mL **	Martinenghi et al., 2020 [37]
CBD	Commercial	*Staphylococcus epidermidis*	ATCC 12228, NRS-60	1–8 µg/mL **	Blaskovich et al., 2021 [11]
Essential oils	Various sources	*Staphylococcus epidermidis*	18Bs	1–16 µg/mL **	Iseppi et al., 2019 [40]
Various terpenes	Commercial	*Staphylococcus epidermidis*	18Bs	8–32 µg/mL **	Iseppi et al., 2019 [40]
CBD	Commercial	*Staphylococcus epidermidis*	18Bs	16 µg/mL **	Iseppi et al., 2019 [40]
CBD	Commercial	*Staphylococcus epidermidis*	ATCC 14990	4 µg/mL **	Abichabki et al., 2022 [31]
CBD	Commercial	*Staphylococcus lugdunensis*	ATCC 43809	4 µg/mL **	Abichabki et al., 2022 [31]
CBG	Commercial	*Staphylococcus mutans*	ATCC 700610	2.5 µg/mL **	Aqawi et al., 2021 [45]
CBD	Commercial	*Streptococcus pneumoniae*	ATCC 33400, ATCC 700677	1–4 µg/mL **	Blaskovich et al., 2021 [11]
CBD	Commercial	*Streptococcus pneumoniae*	ATCC 49619	64 µg/mL **	Abichabki et al., 2022 [31]
CBD	Commercial	*Staphylococcus pyogenes*	ATCC 12344	32 µg/mL **	Abichabki et al., 2022 [31]
CBG	Commercial	*Streptococcus sanguis*	10556	1 µg/mL *	Aqawi et al., 2021 [45]
CBD	Commercial	*Staphylococcus saprophyticus*	ATCC 53050	4 µg/mL **	Abichabki et al., 2022 [31]
CBG	Commercial	*Streptococcus sobrinus*	ATCC 27351	5 µg/mL **	Aqawi et al., 2021 [45]
CBG	Commercial	*Streptococcus salivarius*	ATCC 25975	5 µg/mL **	Aqawi et al., 2021 [45]

* IC50; ** MIC (minimum inhibitory concentration); *** MBC (minimum bactericidal concentration).

**Table 4:** Anti-mycotic efficacy of cannabis-derived molecules and preparations.

Compound	Source	Target	Strain	Efficacy	Reference
Ergost-5-en-3-ol	*C. sativa* (root)	*Cryptococcus neoformans*	ATCC 90113	13.7 μg/mL *	Elhendawy et al., 2019 [26]
Oxygenated derivatives of Δ9-THC and its isomer Δ8-THC	*-*	*Cryptococcus neoformans*	Not noted	2.5–20 μg/mL *	Galal Osman et al., 2018 [39]
CBD	Commercial	*Candida albicans*	SC5314	100 μg/mL ******	Feldman et al., 2019 [46]
Water extract	*C. sativa*, Futura 75	*Candida albicans*	YEPGA 6183	1.4 mg/mL **	Ferrante et al., 2019 [27]
Water extract	*C. sativa*, Futura 75	*Trichophyton interdigitale*	CCC 202–2000	1000 μg/mL **	Orlando et al., 2020 [47]
Water extract	*C. sativa*, Futura 75	*Trichophyton rubrum*	CCC 134–2000	500 μg/mL **	Orlando et al., 2020 [47]

* IC50; ** MIC (minimum inhibitory concentration); ****** MBIC_90_ (minimum biofilm inhibition concentration, 90%).

**Table 5:** Anti-viral efficacy of cannabis-derived molecules and preparations.

Compound	Source	Target	Variant	Microbe	Efficacy	Reference
Δ9-tetrahydrocannabinol (THC)	Lab synthesized	SARS-CoV-2	βCoV/KOR/KCDC03/2020	ssRNA virus	10.25 μM *	Raj et al., 2021 [48]
CBD	Lab synthesized	SARS-CoV-2	βCoV/KOR/KCDC03/2020	ssRNA virus	7.91 μM *	Raj et al., 2021 [48]
Cannabigerolic acid (CBGA)	Commercial	SARS-CoV-2	WA1; B.1.1.7; B.1.351	ssRNA virus	26–37 μg/mL *	Van Breemen et al., 2022 [49]
CBDA	Commercial	SARS-CoV-2	WA1; B.1.1.7; B.1.351	ssRNA virus	11–24 μg/mL *	Van Breemen et al., 2022 [49]
CBD	Commercial	SARS-CoV-2	Not apparent	ssRNA virus	1.27 μM *****	Nguyen et al., 2021 [50]
7-OH-CBD	Commercial	SARS-CoV-2	Not apparent	ssRNA virus	1.27 μM *****	Nguyen et al., 2021 [50]
CBD	Commercial	SARS-CoV-2	WA1/2020	ssRNA virus	1.2 μM *****	Nguyen et al., 2022 [51]
7-OH-CBD	Commercial	SARS-CoV-2	WA1/2020	ssRNA virus	2.6 μM *****	Nguyen et al., 2022 [51]

* IC50; ***** EC50.
